# Kidney outcomes in early adolescence following perinatal asphyxia and hypothermia-treated hypoxic-ischaemic encephalopathy

**DOI:** 10.1007/s00467-022-05705-z

**Published:** 2022-08-17

**Authors:** Katarina Robertsson Grossmann, Liya Vishnevskaya, Sandra Diaz Ruiz, Karolina Kublickiene, Peter Bárány, Mats Blennow, Milan Chromek

**Affiliations:** 1 Department of Clinical Science, Intervention and Technology, Division of Paediatrics, Karolinska Institutet, Stockholm, Sweden; 2 Department of Paediatric Nephrology, Karolinska University Hospital, Stockholm, Sweden; 3 Department of Neonatology, Karolinska University Hospital, Stockholm, Sweden; 4 Department of Radiology, Intervention Unit, Karolinska University Hospital, Stockholm, Sweden; 5 Department of Paediatric Radiology, Karolinska University Hospital, Stockholm, Sweden; 6 Department of Women’s and Children’s Health, Karolinska Institutet, Stockholm, Sweden; 7 Department of Radiology, Lunds University, Lund, Sweden; 8 Department of Clinical Science, Intervention and Technology, Division of Renal Medicine, Karolinska Institutet, Stockholm, Sweden

**Keywords:** Perinatal asphyxia, Acute kidney injury, Hypoxic-ischaemic encephalopathy, Hypothermia treatment, Chronic kidney disease, Long-term outcome

## Abstract

**Background:**

Acute kidney injury (AKI) remains common among infants with hypothermia-treated hypoxic-ischaemic encephalopathy (HIE). Little is known about long-term kidney outcomes following hypothermia treatment. We recently reported that 21% of survivors of hypothermia-treated HIE had decreased estimated glomerular filtration rate (eGFR) based on plasma creatinine in early adolescence. Here, we assessed kidney functions more comprehensively in our population-based cohort of children born in Stockholm 2007–2009 with a history of hypothermia-treated HIE.

**Methods:**

At 10–12 years of age, we measured cystatin C (cyst C) to estimate GFR. Children with decreased cyst C eGFR also underwent iohexol clearance examination. We measured urine-albumin/creatinine ratio, blood pressure (BP) and kidney volume on magnetic resonance imaging. Fibroblast growth factor 23 (FGF 23) levels in plasma were assessed by enzyme-linked immunosorbent assay (ELISA). Outcomes were compared between children with and without a history of neonatal AKI.

**Results:**

Forty-seven children participated in the assessment. Two children (2/42) had decreased cyst C eGFR, for one of whom iohexol clearance confirmed mildly decreased GFR. One child (1/43) had Kidney Disease Improving Global Outcomes (KDIGO) category A2 albuminuria, and three (3/45) had elevated office BP. Subsequent ambulatory 24-h BP measurement confirmed high normal BP in one case only. No child had hypertension. Kidney volume and FGF 23 levels were normal in all children. There was no difference in any of the parameters between children with and without a history of neonatal AKI.

**Conclusion:**

Renal sequelae were rare in early adolescence following hypothermia-treated HIE regardless of presence or absence of neonatal AKI.

**Graphical abstract:**

**Figure:**
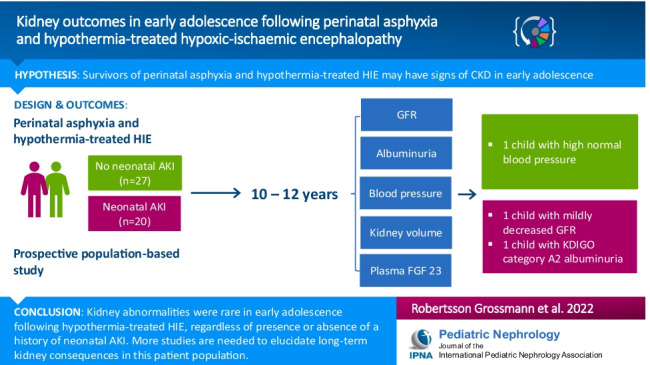
A higher resolution version of the Graphical abstract is available as Supplementary information

**Supplementary Information:**

The online version contains supplementary material available at 10.1007/s00467-022-05705-z.

## Introduction

Perinatal asphyxia severe enough to cause hypoxic-ischaemic encephalopathy (HIE) is often accompanied by multi-organ dysfunction including acute kidney injury (AKI) [1]. In a secondary analysis of the international, multi-centre Assessment of Worldwide Acute Kidney injury Epidemiology in Neonates (AWAKEN) study, 41.3% of 113 new-born infants with HIE suffered neonatal AKI [2]. In this study, however, HIE severity and whether or not infants underwent hypothermia treatment were not reported. The incidence of AKI in infants with hypothermia-treated HIE has been found to be 30–45% in studies using modern staged definitions to define AKI [3–5]. Long-term consequences of neonatal AKI caused by perinatal asphyxia are not well studied. We recently reported that 21% of survivors in our population-based cohort with a history of hypothermia-treated HIE had mildly decreased glomerular filtration rate (GFR) as estimated from creatinine at 10–12 years of age [5]. More is known, however, about long-term consequences of neonatal AKI in other neonatal intensive care (NICU) patient populations. Three recent publications examined chronic kidney disease (CKD) rates in populations born preterm, comparing outcomes between children with and without a history of neonatal AKI. These three single-centre studies reported varying proportions of children with reduced eGFR, ranging from 4% at age 2–3 years in the extremely low birth weight (ELBW) cohort described by Maqsood et al. [6] to 23% at a mean age of 6.6 years in children born below 33 weeks gestational age (GA) in the study by Bruel et al. [7] and 26% at age 3–7 years in a cohort of very low birth weight (VLBW) infants born preterm in the study by Harer et al. [8].

In its early stages, CKD is usually asymptomatic, and early detection is therefore a challenge. Serum creatinine remains the most used biomarker for identification of kidney dysfunction despite its inherent limitations. Assessment of albuminuria and measurement of blood pressure (BP) is common practice in patients who are examined for possible CKD. Kidney size is an important indicator of adequate kidney growth and an indicator for evaluation of kidney disease in children. There are several large cohort studies confirming a strong correlation between kidney volume, body weight and body height in healthy children [9–12]. Many research efforts have been directed towards finding novel biomarkers that may help identify individuals with CKD in its earlier stages to facilitate early intervention. The phosphaturic hormone fibroblast growth factor (FGF) 23 is a novel biomarker of CKD-associated mineral bone disorder (CKD-MBD), which is an important contributor for the increased risk of cardiovascular disease in this patient population [13]. FGF 23 is also associated with hypertension in both paediatric and adult patients [14]. In CKD and transplanted CKD (CKD-T) patients, log FGF23 levels were demonstrated to change significantly at a GFR of 45–38 ml/min/1.73 m^2^ [15]. Another study reported that FGF 23 levels increase significantly at GFR 60–69 ml/min/1.73 m^2^ and below [16]. Paediatric age-specific reference values for FGF 23 have been published [17].

Here, we evaluated long-term kidney outcomes in greater detail in our cohort. We hypothesized that children with a history of hypothermia-treated HIE may have signs of impaired kidney functions in early adolescence, particularly individuals with a history of neonatal AKI. Therefore, our objectives were to (1) estimate or measure GFR and detect albuminuria and high BP, (2) investigate kidney volume using magnetic resonance tomography and (3) to study levels of FGF 23 at age 10–12 years, comparing outcomes in children with and without a history of neonatal AKI.

## Materials and methods

### Study cohort and recruitment

This is a prospective, population-based longitudinal cohort study of children who in infancy underwent whole body hypothermia treatment due to HIE at the Karolinska University Hospital, Stockholm, Sweden, between January 2007 and December 2009, as described previously [5]. Neonatal AKI status according to the Kidney Disease Improving Global Outcomes (KDIGO) definition modified for use in neonatal patients, henceforth referred to as neonatal KDIGO (nKDIGO) [18], had been determined retrospectively by applying both creatinine and urinary output criteria. Exclusion criteria for the present study were a known kidney abnormality/disease or unknown neonatal AKI status.

### Study population source

According to Swedish national guidelines, hypothermia treatment is considered for near-term/term infants with moderate to severe HIE. An asphyxiated near-term/term infant fulfilling any of the following A-criteria: an Apgar score $\le$ 5 at 10 min of age, continued need for resuscitation (including mask ventilation) at 10 min of age, pH < 7.0 within the first 60 min after birth and/or base excess $\le$ − 16 within the first 60 min after birth (in either umbilical cord blood or in an arterial/capillary blood sample) is continuously assessed during the first 60 min after birth for seizures or signs of moderate to severe HIE, defined as the combination of altered level of consciousness, hypotonia OR opisthotonos or abnormal primitive reflexes (B-criteria). Hypothermia treatment is initiated within 6 h after birth in infants fulfilling A- and B-criteria. Infants with known genetic disorders and/or inborn errors of metabolism and/or need for surgery within the first 3 days of life are not offered hypothermia treatment. The two tertiary level NICUs at Karolinska University Hospital serve as regional hypothermia centres where all asphyxiated infants meeting hypothermia treatment criteria are funnelled. Legal guardians of all survivors in the cohort still residing in Sweden were contacted over telephone and invited with their children to participate in the follow-up assessment. They were then sent written information about the study procedures, including children’s age-appropriate information.

### Study procedure

A single standardised study visit was performed at 10–12 years of age. Blood samples were drawn in a standardized manner. Cystatin C (cyst C) was measured by turbidimetry, and estimated GFR (cyst C eGFR) was calculated using the CAPA formula [19]. Analysis was done at the Karolinska University Hospital laboratory immediately after the samples were obtained. In children with decreased cyst C eGFR, GFR was further examined using iohexol clearance soon after the initial assessment. A bolus dose of iohexol was given intravenously, and a first sample was taken immediately thereafter. The concentration–time curves at 180, 210, 240 and 270 min after bolus injection were analysed. Iohexol concentration was measured using ultra high–performance liquid chromatography and photometry.

A morning spot urine sample was measured for albumin and creatinine to generate an albumin/creatinine ratio (UAC). Urine albumin was measured using an immune-turbidimetric method with a measurement interval range of 3–400 mg/L (detection limit 3 mg/L). Urine creatinine was measured using an enzymatic photometric method with a measurement interval of 0.1–54 mmol/L (detection limit 0.1 mmol/L). Both were analysed using the automation platform Roche Cobas 8000 instrument module c701 in accordance with the manufacturer’s instructions. In case of elevated UAC, a second sample was requested approximately 3 months after the initial sample to confirm presence of persistent albuminuria.

Height and weight were measured and plotted on Swedish national growth charts based on the World Health Organisation growth charts from 2006 [20]. Oscillometric BP-measurements were performed three times after 10 min of rest in supine position using a size-appropriate cuff on the arm. The average systolic and diastolic BPs were compared with the age-, height-, and sex-specific BP nomograms. BP was analysed in accordance with the Swedish national guidelines for management of hypertension in children and adolescents from 2020 (www.nefro.barnlakarforeningen.se) which are based on the 2016 European Society of Hypertension guidelines for the management of high blood pressure in children and adolescents [21]. Children with elevated office BP were further examined with a 24-h ambulatory BP-measurement (ABPM) (SpaceLabs, USA).

Magnetic resonance imaging (MRI) of the kidneys was acquired using a Sigma 3.0 Tesla MR scanner (Discovery MR750, General Electric Healthcare, USA) at MR-Centrum, Karolinska Institutet, Solna. T2-weighted, fat-suppressed images with enhanced reconstruction (PROPELLER) were acquired with 20 slices per sequence (slice thickness 3.0 mm, TE 84.8 ms, flip angle 140°, TR 11.639 s, FOV 30 cm) using a head-and-neck CTL spine coil. Coronal plane images were obtained and used for kidney volume calculation. Any morphological renal abnormalities were noted. The semi-automatic segmentation method based on manual contour delineation with contour interpolation was used within the MM Reading protocol of Syngo.Via software (Siemens, Germany) [22, 23]. Sinus fat and renal pelvis were excluded from the segmented area of renal parenchyma. All volume quantifications were carried out by a single experienced radiologist blinded to the clinical data. The length of each kidney was measured on coronal plane images. Kidney length and volume (individual and combined) were then compared to sonographic growth charts based on measurements of kidney length and use of ellipsoid formula method to calculate kidney volume [24].

FGF 23 (pg/ml) was analysed by second-generation human sandwich enzyme-linked immunosorbent assay (ELISA, Quidel, Ireland, detection limit 0.0015 ng/mL), according to the manufacturer’s instructions at the NEO laboratory at Karolinska Institutet, Huddinge.

### Ethics statement

The study was approved by the Ethics Review Board in Sweden (2019–01447). Written consent from children’s legal guardians was obtained prior to participation.

### Outcomes

Our primary outcomes were decreased GFR, albuminuria, and hypertension. Decreased GFR was defined as GFR < 90 ml/min/1.73 m^2^ as estimated by cystatin C (cyst C eGFR) or measured by iohexol clearance [25]. Category of albuminuria was classified according to the KDIGO definition as follows: normal to mildly increased (A1) defined as a  UAC < 3 mg/mmol, moderately increased (A2) defined as a UAC 3–30 mg/mmol and severely increased (A3) as a UAC > 30 mg/mmol [25]. Hypertension was defined as a systolic or diastolic BP equal to or above the 95th percentile for age, height and sex on ABPM or a history of hypertension currently being treated with antihypertensive medication. High normal BP was defined as a systolic or diastolic BP above the 90th but below the 95th percentile for age, height and sex on ABPM [26]. Secondary outcomes were kidney volume on MRI and plasma levels of FGF 23. Kidney volume was compared to nomograms based on ultrasonographical approximation of kidney volume based on kidney length using the ellipsoid formula [24]. Furthermore, individual total kidney volume Z-scores were calculated. Individual FGF 23 levels in plasma were compared to age-specific percentiles published by Fischer et al. [17]. For this purpose, values were converted from pg/ml to RU/ml (factor $\times 0.5$).

### Statistical analysis

Variables were tested for normality with the Kolmogorov–Smirnoff test. Data are presented as means with standard deviation (SD) and 95% confidence intervals (CIs) for normally distributed numerical variables and as medians with interquartile range (IQR) for numerical variables that are not normally distributed. To compare outcomes on a group level between children with and without a history of neonatal AKI, univariate analysis with Fischer’s exact test was used to compare dichotomous, nominal variables, whereas a *t*-test was used to compare normally distributed, numerical variables, and the Mann–Whitney test was used for comparison of non-normally distributed continuous numerical variables. A *p*-value < 0.05 was considered statistically significant for all analyses. All analyses were performed using GraphPad Prism version 9.3.1 for MacOS (San Diego, CA, U.S.A.).

## Results

### Index neonatal data

Relevant clinical data from the NICU hospital stay including sex, gestational age at birth, birth weight, need for full cardio-pulmonary resuscitation, HIE severity, neonatal AKI status according to the nKDIGO definition [18], duration of mechanical ventilation, length of hospital stay, need for kidney support therapy and all-cause mortality have been previously reported [5]. Neonatal patient characteristics are summarised in Supplementary Table 1.

### Kidney outcome characteristics

**Fig. 1:**
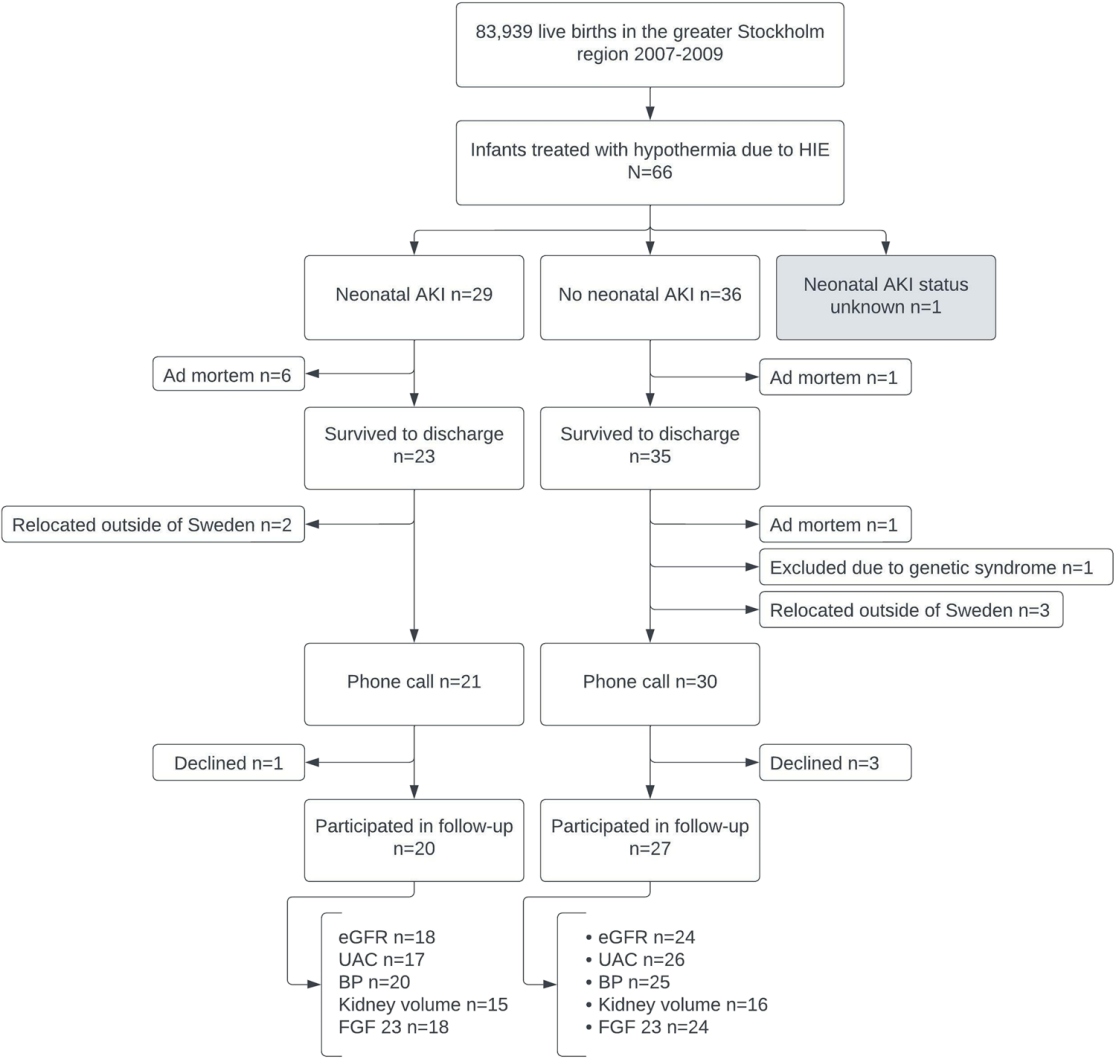
Flow diagram that outlines the available study population (NeoCool cohort) and those who participated in the present study of long-term kidney outcomes following hypothermia-treated HIE. AKI, acute kidney injury; BP, blood pressure; Cyst C eGFR, cystatin C-estimated GFR; FGF, fibroblast growth factor; HIE, hypoxic-ischaemic encephalopathy

At 10–12 years of age, 48 children were enrolled in the follow-up study. Eight children had died, the vast majority already prior to discharge from the neonatal intensive care unit. Five families had relocated outside of Sweden, and four families actively declined participation at this time. Neonatal AKI status was known for final analysis in 47 children (Fig. 1). Neonatal AKI had been diagnosed in 43% (20/47) of them: 34% (16/47) stage 1 AKI and 9% (4/47) stage 3 AKI. No children taking part in this follow-up study had a history of stage 2 AKI. The remaining 57% had not suffered neonatal AKI. Forty-two children agreed to have blood samples taken. Forty-three children provided a morning urine sample. BP could be measured in 45 children during the follow-up visit. In the case of two children with cerebral palsy and plenty of involuntary movement, it was not possible to obtain reliable BP measurements. Thirty-one children agreed to and were able to complete an MRI examination.

Two children (2/42), one with and one without a history of neonatal AKI, had mildly decreased cyst C eGFR. They had cyst C eGFR of 85 and 87 ml/min/1.73 m^2^, respectively. These two children were further examined using iohexol clearance. Iohexol clearance confirmed mildly decreased GFR (88 ml/min/1.73 m^2^) in one of the children (Table 1). This child had needed kidney support therapy (continuous veno-venous haemodiafiltration) for a period of 3 days during the neonatal period, thus fulfilling the nKDIGO criteria for stage 3 AKI. One child (1/43) had KDIGO category A2 albuminuria. This child also had a history of stage 3 AKI. The remaining 42 children had KDIGO category A1 albuminuria with a UAC < 3 mg/mmol. No child had KDIGO category A3 albuminuria (Table 1). Seven per cent of children (3/45) were found to have elevated office BP. These children were further examined with ambulatory 24-h BP-measurement which revealed high normal BP in the case of one child without a history of neonatal AKI, while the other two children had normal BP. No child had hypertension (Table 1).

**Table 1:** Summary of kidney outcome characteristics at age 10–12 years according to neonatal AKI status

Outcome variable	History of neonatal AKI (*n* = 20)	No history of neonatal AKI (*n* = 27)
**GFR:**		
• Cyst C eGFR < 90 ml/min/1.73 m^2^	• 1/18	• 1/24
• GFR < 90 ml/min/1.73 m^2^ confirmed by iohexol clearance	• 1/1	• 0/1
**Urine albumine/creatinine ratio:**		
• KDIGO category A1 albuminuria	• 16/17	• 26/26
• KDIGO category A2 albuminuria	• 1/17	• 0/26
• KDIGO category A3 albuminuria	• 0/17	• 0/26
**BP:**		
• Elevated office BP	• 1/20	• 2/25
• Hypertension confirmed by 24-h ABPM	• 0/1	• 0/2
• High normal BP confirmed by 24-h ABPM	• 0/1	• 1/2
**Kidney volume (total):**		
• Decreased kidney volume compared to nomograms	• 0/15	• 0/16
• Mean Z-score (SD, 95% CI)	• − 0.08 (1.18, − 0.74 to 0.57)	• 0.09 (0.79, − 0.33 to 0.51)
**Plasma FGF 23 in pg/ml:**		
• FGF 23 > 90th percentile for age	• 0/18	• 0/24
• Median (IQR)	• 37.9 (29.73 to 52.55)	• 34.85 (26.38 to 42.88)

KDIGO categories of albuminuria: normal to mildly increased (A1) defined as a urine albumin/creatinine ratio < 3 mg/mmol, moderately increased (A2) defined as a urine albumin/creatinine ratio 3–30 mg/mmol and severely increased (A3) as a urine albumin/creatinine ratio > 30 mg/mmol [25]. Hypertension was defined as a systolic or diastolic BP equal to or above the 95th percentile for age, height and sex on ABPM, or a history of hypertension currently being treated with antihypertensive medication. High normal BP was defined as a systolic or diastolic BP above the 90th but below the 95th percentile for age, height and sex on ABPM [26]. Kidney volume compared to nomograms based on ultrasonographical approximation of kidney volume based on kidney length using the ellipsoid formula [24]. Individual FGF 23 levels in plasma were compared to age-specific percentiles published by Fischer et al. [17]. No significant difference was detected between the two groups for any of the variables

*AKI* acute kidney injury, *ABPM* ambulatory blood pressure measurement, *BP* blood pressure, *Cyst C eGFR* cystatin C-estimated GFR, *KDIGO* Kidney Disease Improving Global Outcomes, *SD* standard deviation, *CI* confidence interval, *FGF* fibroblast growth factor, *IQR* interquartile range

### Kidney volume

**Fig. 2:**
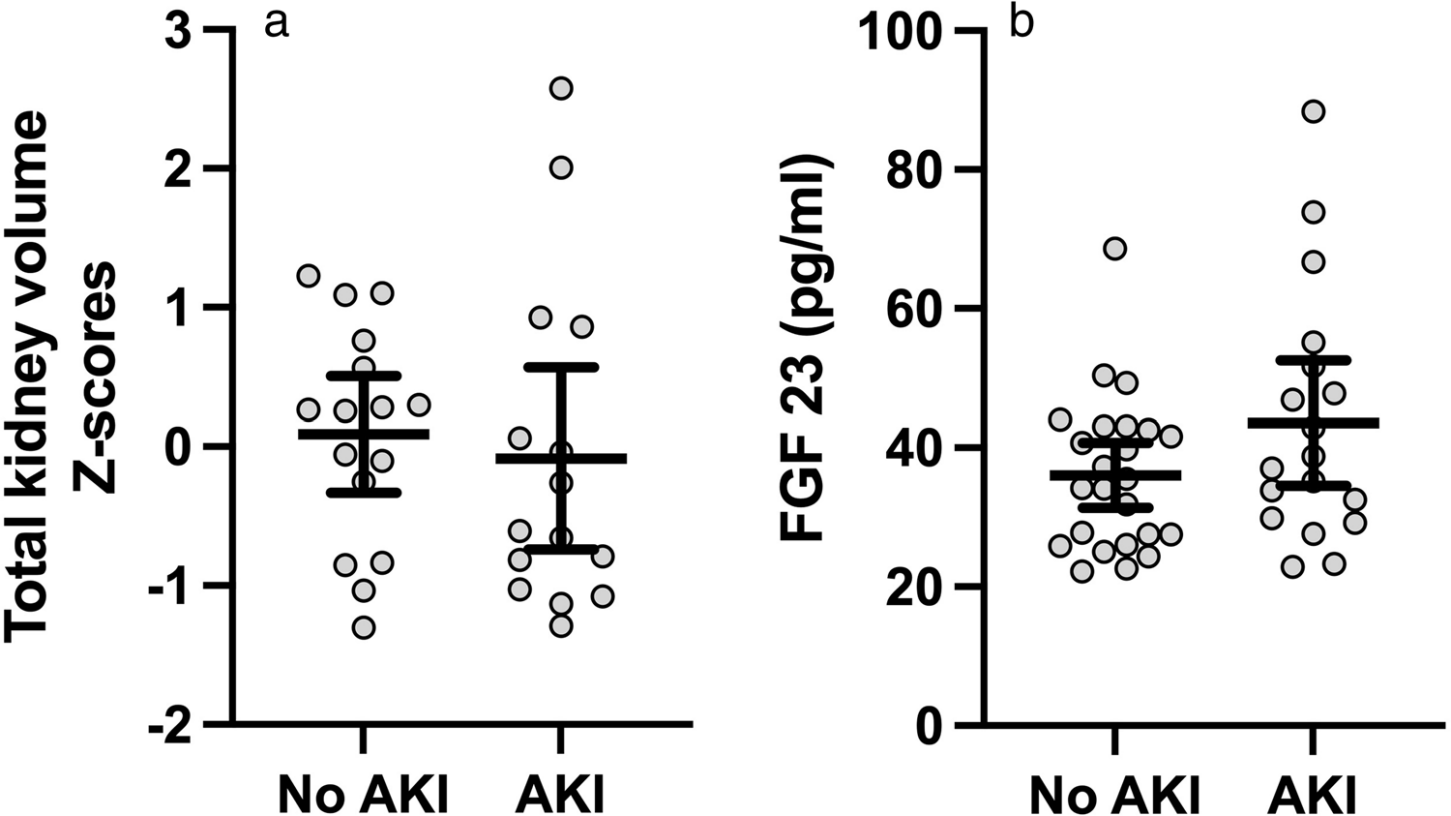
**a** Total kidney volume Z-scores at age 10–12 years according to neonatal AKI-status, shown with mean and 95% CI bars. **b** Individual FGF 23 levels in pg/ml at age 10–12 years according to neonatal AKI-status, shown with median and IQR bars. AKI, acute kidney injury; FGF, fibroblast growth factor; IQR, interquartile range. No significant difference was detected between the two groups for either variable

Thirty-one children with known neonatal AKI status agreed to and were able to complete an MRI examination to assess kidney volume. Fifteen of them (48%) had a history of neonatal AKI. One child was found to have a unilateral hypoplastic kidney with compensatory enlargement of the contralateral kidney resulting in normal total kidney volume. Another patient had suspected minor parenchymal scarring bilaterally not affecting the total kidney volume. This child had no history of upper urinary tract infection. No child had reduced total kidney volume when compared to nomograms based on ultrasonographical approximation of kidney volume based on kidney length using the ellipsoid formula [24]. There was no difference in mean kidney volume Z-score between children with a history of neonatal AKI (– 0.08, SD 1.18, 95% CI – 0.74 to 0.57) compared to those without (0.09, SD 0.79, 95% CI – 0.33 to 0.51) (Table 1). Individual kidney volume Z-scores according to neonatal AKI status are illustrated in Fig. 2a.

#### FGF23

Median FGF 23 was 36.25 pg/ml (IQR 27.5–44.73 pg/ml). No child had an FGF 23 value above the 90th percentile for age [17, 27]. We found no difference when comparing FGF 23 levels among children with a history of neonatal AKI (median 37.9 pg/ml, IQR 29.73 to 52.55 pg/ml) to those without a history of neonatal AKI (median 34.85 pg/ml, IQR 26.38 to 42.88 pg/ml) (Table 1). Individual FGF 23 levels according to neonatal AKI status are illustrated in Fig. 2b.

## Discussion

To our knowledge, this is the first report exploring long-term kidney outcomes in detail among young adolescents with a history of perinatal asphyxia and hypothermia-treated HIE. In our cohort, decreased GFR, albuminuria, hypertension/elevated BP, reduced kidney volume and/or increased plasma level of FGF 23 were rare at the age of 10–12 years.

Our previous findings suggested that 21% of survivors in our cohort had decreased eGFR as estimated with the Schwartz–Lyon equation. Interestingly, we found no difference in the incidence of mildly decreased eGFR when comparing children with and without a history of neonatal AKI [5]. The phenotype in CKD is known to be variable; chronic kidney impairment can manifest in the form of decreased GFR or hyperfiltration, proteinuria, tubular dysfunction, urinary sediment abnormalities, hypertension and/or imaging abnormalities persisting for at least 3 months [25, 28]. This prompted us to assess kidney functions in greater detail in our cohort, using a multimodal approach. Our more in-depth assessment of kidney functions revealed mildly decreased cyst C eGFR only in two children (5%), and subsequent iohexol clearance examination confirmed mildly decreased GFR only in one child (2.5%). Although eGFR based on serum creatinine is widely used, it can in certain populations underestimate or overestimate GFR, which has been discussed in a large cohort of apparently healthy adolescents [29]. The most commonly used Schwartz–Lyon equation was derived from paediatric CKD patients. It has been suggested that equations developed in populations with decreased GFR underestimate GFR in individuals without kidney disease, both among adults, adolescents and children [30], thus further supporting the need of more comprehensive assessment of kidney function. Only one child was found to have KDIGO category A2 albuminuria; all other children had a UAC < 3 mg/mmol. No child had hypertension. One child had high normal BP. Total kidney volume and FGF 23 levels were normal in all children for whom data was available. We found no difference in any of the parameters when comparing children with and without a history of neonatal AKI.

AKI is a frequent complication among critically ill patients treated in the NICU [31]. One NICU population at special risk of AKI are infants with post-asphyxial HIE. Studies from the pre-hypothermia treatment era, using various AKI definitions, reported AKI incidences between 42 and 70% [32–34]. In our cohort, 45% of infants had suffered AKI as defined according to nKDIGO [5], which is somewhat higher compared to previously reported incidences in studies by Bozkurt et al. and Selewski et al. [3, 4]. Both these studies used the modified Acute Kidney Injury Network (AKIN) definition. A secondary analysis from the multicentre Assessment of Worldwide Acute Kidney injury Epidemiology in Neonates (AWAKEN) initiative on 113 infants with HIE found that 41.6% of patients suffered AKI. This study, however, did not report HIE severity or if the participant had undergone hypothermia treatment, hampering comparisons with other studies [2]. Thus, it remains unclear if therapeutic hypothermia reduces the risk for AKI in this patient group. Hitherto, long-term outcome studies in survivors of hypothermia-treated HIE have largely focused on neurologic, cognitive, and developmental outcomes. Interestingly, Sarkar et al. found that neonatal AKI was independently associated with hypoxic-ischaemic lesions on MRI of the brain at 7–10 days of life [35], and Cavallin et al. reported that neonatal AKI was associated with increased likelihood of adverse outcome, defined as death or disability according to the Griffiths Developmental Scales at 24 months of age [36]. In the latter study, the positive predictive value (1.00, 95% CI 0.71–1.00) and specificity (1.00, 95% CI 0.88–1.00) of AKI were good, while the negative predictive value (0.41, 95% CI 0.30–0.52) and sensitivity (0.19, 95% CI 0.11–0.32) were poor.

Several studies on long-term kidney outcomes have been done in other NICU populations. Sanderson et al. found that in a cohort of children born before 28 weeks GA, 50% had one or more abnormalities associated with CKD (reduced kidney volume, category A2 albuminuria and/or elevated BP) at 15 years of age [37]. In a retrospective study, Maqsood et al. investigated kidney outcomes 2–3 years after discharge from the NICU in a cohort of ELBW infants with and without a history of neonatal AKI, reporting CKD in 4% of patients and no correlation with neonatal AKI status [6]. The Follow-up of Acute kidney injury in Neonates during Childhood Years (FANCY) study by Harer et al. assessed kidney dysfunction using a multimodal approach in a cohort of VLBW infants born preterm with and without a history of neonatal AKI. They found that at a median age of 5 years, 26% of children in their cohort had GFR < 90 ml/min/1.73 m^2^ as estimated by cyst C. Children with a history of neonatal AKI had a 4.5 times higher risk of kidney dysfunction [8]. Bruel et al. investigated the role of neonatal AKI on long-term kidney health in a cohort of infants born at less than 33 weeks gestational age, defining AKI based on varying creatinine cut-off levels depending on gestational age. When compared to a propensity-matched group of children born preterm without a history of neonatal AKI, children with a history of neonatal AKI were demonstrated to have smaller kidneys at a mean age of 6.6 years. In this cohort, 23% of children had GFR < 90 ml/min/1.73 m^2^ as estimated from serum creatinine, and 11% had microalbuminuria. There was no difference in eGFR, microalbuminuria or pulse wave velocity between the AKI and the no AKI groups. Kidney volume was significantly lower in the AKI group [7]. A recent systematic review on the effect of prematurity on long-term kidney health found that prematurity is likely linked to an increased risk for renal dysfunction and elevated/high BP in childhood and into early adulthood. Extremely premature birth conferred a threefold increase in the risk of CKD, whereas premature birth conferred a twofold increase in the risk of CKD [38].

New-born infants needing cardiac repair surgery constitute another NICU population at high risk of AKI [39]. In a study published in 2020, Huynh et al. reported that 17% of subjects had CKD, and 30% had hypertension at a median follow-up age of 6 years. An additional 15% were found to have elevated BP. Interestingly, the same group found that cardiac surgery–associated AKI was not associated with CKD or hypertension [40]. In comparison, the prevalence of renal abnormalities in our cohort was much lower. Similarly to Huynh et al., we did not find a difference in the incidence of decreased eGFR as estimated from creatinine and the Schwartz–Lyon equation [5] or by other methods in the current study when comparing children with and without history of AKI.

The usefulness of kidney volume as a marker of kidney function is widely discussed [41]. Several studies suggest the use of relative total kidney volume as a non-invasive marker of kidney function in both adult and paediatric populations [42–44]. Current kidney size nomograms are still based on kidney lengths [24]. Historically, calculation of kidney volume by ellipsoid formula method on ultrasound has been the gold standard [9, 24, 41]. This method has been shown to underestimate kidney volume compared to computer tomography or MRI segmentation [45]. Using MRI to measure kidney size and volume is not a common practice; however, the segmentation volumetry method which we employed is acceptably time-consuming while it gives more accurate estimation of kidney volume than ellipsoid formula calculation [22, 23]. Currently, there are no existing normal ranges of kidney volume based on CT- or MRI-based segmentation methods in paediatric populations. Park et al. evaluated the relationship between anthropometric indices and kidney length and volume as measured on CT in 272 Korean children without kidney disease, showing that body surface area has the strongest correlation with kidney volume. As these authors determined the mean kidney size for each age and height group, they suggested that their results may serve as normative standards for assessment of renal growth, but their findings have not yet been validated in other populations [46]. In our cohort, no child had reduced total kidney volume which contrasts with findings reported by Bruel et al. in a cohort of children born preterm with a VLBW [7]. This may in part be explained by the fact that the children in our cohort were born at a GA when nephrogenesis is completed.

Although renal abnormalities were rare in early adolescence among the children in our cohort, long-term follow up is still warranted. This was also highlighted by Sanna-Cherchi et al. in a follow up study of 312 patients with congenital anomalies of the kidney and urinary tract until 30 years of age, where renal deterioration could not be detectable until late adolescence in patients with kidney anomalies other than posterior urethral valves or bilateral hypodysplasia [47].

Frequently, there is a delay in the diagnosis as CKD is initially asymptomatic [48]. A delay in identifying CKD in its early stages is associated with a more rapid disease progression and premature mortality in patients with kidney failure [48]. One of the most important causes of death in paediatric CKD is cardiovascular disease [49], for which CKD-MBD is a major contributor [13]. FGF 23 levels in plasma have been demonstrated to increase earlier than serum creatinine in paediatric patients with CKD [15, 16]. As we had previously found that 21% of survivors in our cohort had decreased eGFR based on creatinine at age 10–12 years, we hypothesized that the children in our study might have increased levels of FGF 23 as an early marker of CKD. We found no child with FGF 23 above the 90th percentile for age [17]. Moreover, there was no difference in FGF 23 levels when comparing children with a history of neonatal AKI to those without. As none of the participants in our study had GFR below 70 ml/min/1.73 m^2^, it is not surprising that FGF 23 levels were within normal range.

Strengths of our study include its population-based and prospective design as well as a multimodal approach to evaluate kidney functions. Our study is not without limitations. It is a single-centre study, and the sample size is likely too small to detect differences in subgroups of participants. The vast majority of children in our cohort with a history of neonatal AKI had suffered stage 1 AKI as per the nKDIGO definition, which could in part explain the low proportion of children with renal sequelae at the time of follow-up assessment. It has, however, been argued that the current nKDIGO definition may not be sensitive enough to adequately identify all infants who suffer AKI during the first week of life [50]. It was not possible for all participating children to complete all examinations in the study protocol. Outcome measures were determined at a single visit, which could be argued may not reflect a chronic trajectory in this sample. Dixon 3D sequences could have further improved MRI measurement reliability.

To the best of our knowledge, we are the first to evaluate long-term kidney outcomes after hypothermia-treated HIE. In summary, the incidence of kidney abnormalities was low in our cohort of young adolescents with a history of hypothermia-treated HIE, regardless of the presence or absence of neonatal AKI. We therefore suggest that larger and long-term studies are needed to elucidate the possible kidney consequences in this patient population.

## Supplementary Information

Below is the link to the electronic supplementary material.

Supplementary file1 Supplemental Table 1. Summary of patient characteristics from the neonatal period, presented in absolute numbers (with percentage withing brackets) or as medians (with IQR within brackets) as appropriate. Total number of patients N=65. Abbreviations: HIE, hypoxic-ischaemic encephalopathy; AKI, acute kidney injury; nKDIGO, Kidney Disease Improving Global Outcomes definition of AKI modified for use in neonatal patients, IQR, interquartile range. (DOCX 15 KB)

Graphical Abstract (PPTX 64 KB)
