# Supplementary material for: Kidney outcomes in early adolescence following perinatal asphyxia and hypothermia-treated hypoxic-ischaemic encephalopathy
Source: Pediatr Nephrol. 2022 Aug 17;38(4):1205–14. doi: 10.1007/s00467-022-05705-z (PMC9925534; doi:10.1007/s00467-022-05705-z)
Supplement: Supplementary file 1 — Supplementary file1 Supplemental Table 1. Summary of patient characteristics from the neonatal period, presented in absolute numbers (with percentage withing brackets) or as medians (with IQR within brackets) as appropriate. Total number of patients N=65. Abbreviations: HIE, hypoxic-ischaemic encephalopathy; AKI, acute kidney injury; nKDIGO, Kidney Disease Improving Global Outcomes definition of AKI modified for use in neonatal patients, IQR, interquartile range. (DOCX 15 KB) [file 467_2022_5705_MOESM1_ESM.docx]

Supplemental table 1.

| **Patient characteristics** | |
| --- | --- |
| Sex (boys/girls) | 31/34 |
| Gestational age at birth in weeks + days, median (IQR) | 40+2 (38+6 – 41+2) |
| Birth weight in grams, median (IQR) | 3498 (3145 – 4055) |
| Apgar at 5 min, median (IQR) | 3 (0 – 4) |
| Apgar at 10 min, median (IQR) | 4 (2 – 6) |
| Sarnat stage of HIE:   - Stage 1 - Stage 2 - Stage 3 | 4 (6%)  50 (77%)  11 (17%) |
| Stage of neonatal AKI:   - No AKI - Any stage - Stage 1 - Stage 2 - Stage 3 | 36 (55%)  29 (45%)  22 (34%)  2 (3%)  5 (8%) |
| AKI (any stage) as per nKDIGO definition by urinary output and plasma creatinine criteria:   - Urinary output only - Plasma creatinine only - Both criteria | 13 (20%)  7 (11%)  9 (14%) |
| Neonatal mortality | 7 (11%) |
| Total all-cause mortality | 8 (12%) |
