# Supplementary material for: Kidney outcomes in early adolescence following perinatal asphyxia and hypothermia-treated hypoxic-ischaemic encephalopathy
Source: Pediatr Nephrol. 2022 Aug 17;38(4):1205–14. doi: 10.1007/s00467-022-05705-z (PMC9925534; doi:10.1007/s00467-022-05705-z)
Supplement: Supplementary file 2 — Graphical Abstract (PPTX 64 KB) [file 467_2022_5705_MOESM2_ESM.pptx]

## Slide 1
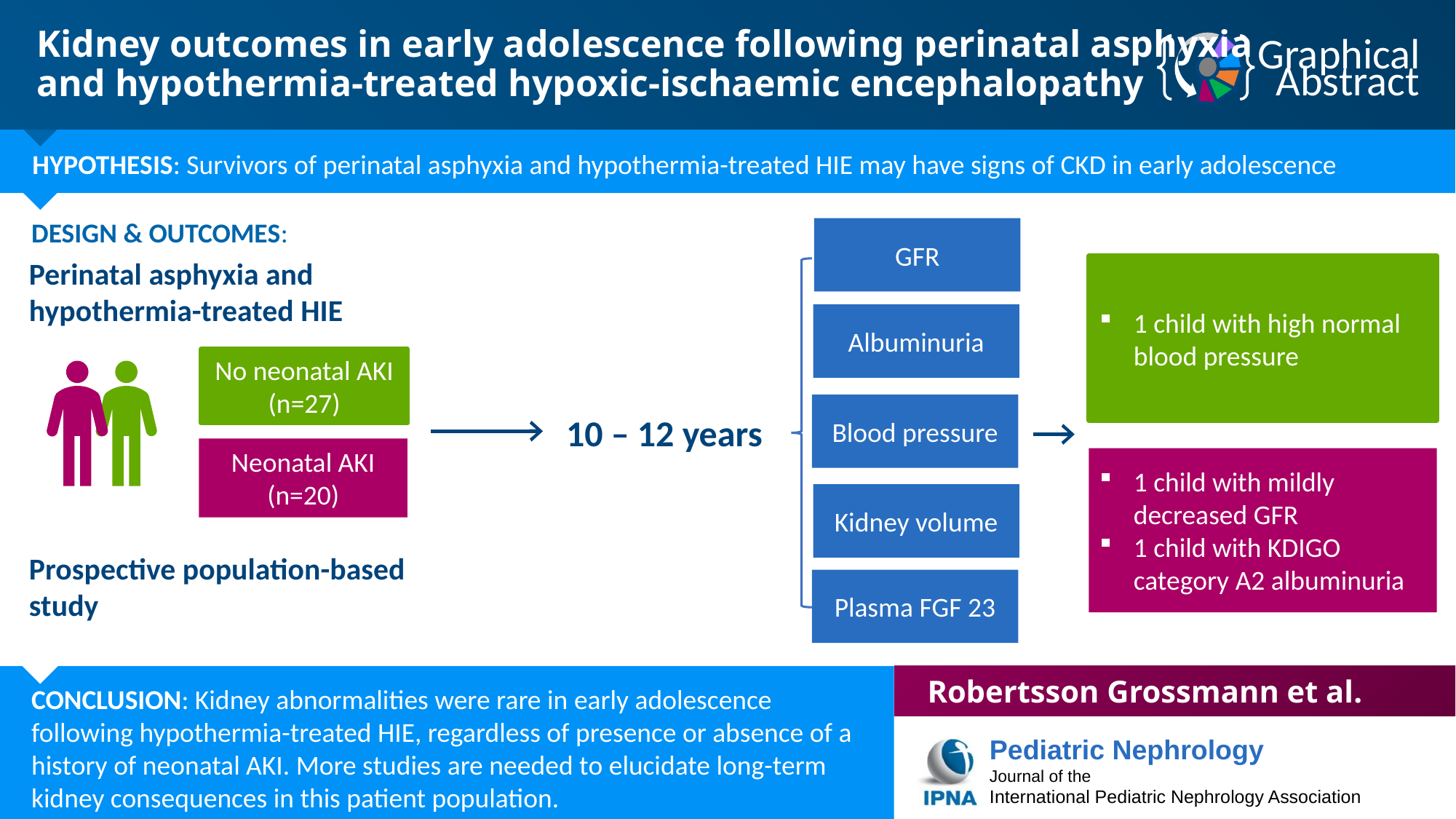

Kidney outcomes in early adolescence following perinatal asphyxia
and hypothermia-treated hypoxic-ischaemic encephalopathy
HYPOTHESIS: Survivors of perinatal asphyxia and hypothermia-treated HIE may have signs of CKD in early adolescence
DESIGN & OUTCOMES:
GFR
Perinatal asphyxia and hypothermia-treated HIE
1 child with high normal blood pressure
Albuminuria
No neonatal AKI (n=27)
Blood pressure
10 – 12 years
Neonatal AKI (n=20)
1 child with mildly decreased GFR
1 child with KDIGO category A2 albuminuria
Kidney volume
Prospective population-based study
Plasma FGF 23
Robertsson Grossmann et al. 2022
CONCLUSION: Kidney abnormalities were rare in early adolescence following hypothermia-treated HIE, regardless of presence or absence of a history of neonatal AKI. More studies are needed to elucidate long-term kidney consequences in this patient population.
